# Transcutaneous Electrical Nerve Stimulation (TENS) Alleviates Brain Ischemic Injury by Regulating Neuronal Oxidative Stress, Pyroptosis, and Mitophagy

**DOI:** 10.1155/2023/5677865

**Published:** 2023-04-17

**Authors:** Zixuan Tan, Fang Dong, Linyu Wu, Yashuo Feng, Min Zhang, Feng Zhang

**Affiliations:** ^1^Department of Rehabilitation Medicine, The Third Hospital of Hebei Medical University, Shijiazhuang 050051, China; ^2^Department of Clinical Laboratory Medicine, The Third Hospital of Hebei Medical University, Shijiazhuang 05005, China; ^3^Department of Pathophysiology, Hebei Medical University, Shijiazhuang 050051, China; ^4^Hebei Key Laboratory of Critical Disease Mechanism and Intervention, Shijiazhuang 050051, China

## Abstract

**Background:**

As a noninvasive treatment, transcutaneous electrical nerve stimulation (TENS) has been utilized to treat various diseases in clinic. However, whether TENS can be an effective intervention in the acute stage of ischemic stroke still remains unclear. In the present study, we aimed to explore whether TENS could alleviate brain infarct volume, reduce oxidative stress and neuronal pyroptosis, and activate mitophagy following ischemic stroke.

**Methods:**

TENS was performed at 24 h after middle cerebral artery occlusion/reperfusion (MCAO/R) in rats for 3 consecutive days. Neurological scores, the volume of infarction, and the activity of SOD, MDA, GSH, and GSH-px were measured. Moreover, western blot was performed to detect the related protein expression, including Bcl-2, Bax, TXNIP, GSDMD, caspase-1, NLRP3, BRCC3, HIF-1*α*, BNIP3, LC3, and P62. Real-time PCR was performed to detect NLRP3 expression. Immunofluorescence was performed to detect the levels of LC3.

**Results:**

There was no significant difference of neurological deficit scores between the MCAO group and the TENS group at 2 h after MCAO/R operation (*P* > 0.05), while the neurological deficit scores of TENS group significantly decreased in comparison with MCAO group at 72 h following MACO/R injury (*P* < 0.05). Similarly, TENS treatment significantly reduced the brain infarct volume compared with the MCAO group (*P* < 0.05). Moreover, TENS decreased the expression of Bax, TXNIP, GSDMD, caspase-1, BRCC3, NLRP3, and P62 and the activity of MDA as well as increasing the level of Bcl-2, HIF-1*α*, BNIP3, and LC3 and the activity of SOD, GSH, and GSH-px (*P* < 0.05).

**Conclusions:**

In conclusion, our results indicated that TENS alleviated brain damage following ischemic stroke via inhibiting neuronal oxidative stress and pyroptosis and activating mitophagy, possibly via the regulation of TXNIP, BRCC3/NLRP3, and HIF-1*α*/BNIP3 pathways.

## 1. Introduction

As one of the major causes of high mortality and morbidity rates worldwide, stroke is a multifactorial and devastating disease, which can be broadly classified into two forms: ischemic stroke and hemorrhagic stroke [1]. To sum up, more than 70% of stroke cases are ischemic stroke in the world, and this percentage is reaching nearly 86% in the United States of America [2–5]. Ischemic stroke is usually occurred due to the embolic or thrombotic occlusion in cerebral arteries, which leads to permanent physical and neurological disabilities, contributing to high expenditure on health care annually. Currently, two major timely revascularization treatments, including endovascular thrombectomy and intravenous thrombolysis, are recommended to apply in the acute phase of ischemic stroke according to the present clinical guidelines [6–8]. However, they have certain limitations, such as the narrow therapeutic window, multiple contraindications, and strict eligibility standard [9, 10]. Despite progress in the investigation of the pathogenesis of cerebral ischemia, the safe and reliable therapeutic methods are still very limited [11–13]. Thus, a safe and beneficial therapeutic method for stroke is urgently needed in clinical settings.

Electroacupuncture (EA) combines electrical stimulation with traditional acupuncture, which is an extension technique based on acupuncture [14]. EA is widely applied in both clinical settings and experimental research for stroke treatment [15]. Our previous studies and related researches had shown that EA intervention effectively reduced the cerebral damage following ischemic stroke via modulating various pathophysiological processes, including apoptosis, inflammation, autophagy, and analgesia [15–17]. However, EA treatment is invasive with the risks of bleeding and infection, and some stroke patients cannot bear the pains of puncturing. Transcutaneous electrical nerve stimulation (TENS) is a technique of electrical stimulation, which can produce perceptible sensations by electrodes in contact with skin [18]. TENS is used widely in clinical practice in various types of hospitals and medical institutions because of its simplicity, good safety, and noninvasion [19]. Currently, the analgesic effects of TENS on multiple neuropathic pains are extensively studied, such as cancerous pain and musculoskeletal pain [19, 20]. However, TENS has only been proved to exert beneficial effects on patients with ischemic stroke in late stages in clinical trials. For instance, Chen et al. demonstrated that bilateral TENS combined with task-oriented training effectively improved motor recovery of upper limb following stroke [21]. Moreover, a systematic review and meta-analysis demonstrated that TENS was related to the improvement of spasticity and promoted walking speed and static balance in stroke patients [22]. Actually, increasing evidence supports early rehabilitation interventions after stroke are of important significance, which can rebuild brain function, accelerate the average cerebral blood flow, and promote collateral circulation formation, thus improving the neurological function of patients [23–27]. Therefore, clarifying whether and how TENS exerts beneficial effects at the early stage of ischemic stroke will provide theoretical basis and promote the extensive application of TENS in clinic.

Following the obstruction of brain arteries by an in situ thrombosis or embolus, cerebral ischemia occurs and causes irreversible neuronal dysfunction and a cascade of pathological responses, comprising apoptosis, oxidative stress, neuroinflammation, and autophagy [28]. Oxidative stress plays a key role in the fundamental pathological progression of brain injury following ischemic stroke. Oxidative stress occurs when the inherent antioxidant capacity to counteract reactive oxygen species (ROS) is insufficient and cannot maintain the endogenous redox balance [29, 30]. Oxidative stress can result in cytotoxicity due to oxidative damage of nucleic acids, protein, and lipids with detrimental effects on the function and structure of brain tissues [31]. As a primary antioxidant system, thioredoxin (TRX) system is essential to keep the intracellular redox homeostasis, and thioredoxin-interacting protein (TXNIP) is an endogenous suppressor of TRX [32, 33]. Tian et al. reported that TXNIP increased the levels of oxidative stress in ischemic stroke, and inhibition of TXNIP conferred the neuroprotection against ischemic brain damage. On the other hand, neuroinflammation is a vital event contributing to brain injury after stroke [34]. In the innate immune system, inflammasomes are the key regulators of neuroinflammatory response in ischemic stroke, such as the nod-like receptor pyrin domain-containing 3 (NLRP3) inflammasome [35]. The increase of NLRP3 inflammasomes stimulates pro-caspase-1, which causes the aggravation of pyroptosis, a form of inflammatory programmed cell death [36, 37]. The suppression of NLRP3 obviously reduces cerebral ischemic injury, protecting against neuronal cell death in both in vivo and in vitro settings, which may be an effective strategy for treating stroke [38]. Moreover, BRCA1eBRCA2-containing complex subunit 3 (BRCC3) is a critical modulator of NLRP3 activity through increasing its deubiquitination and making NLRP3 a characterized substrate for the cytosolic BRCC3-containing BRISC complex [39]. However, the precise alteration in BRCC3 levels and whether TENS exerts counteracting effects on oxidative stress and inflammasomes by TXNIP and BRCC3/NLRP3 signaling have barely been investigated in ischemic stroke.

Autophagy is referred to a regulatory mechanism for recycling and degrading cellular constituents and managing protein quality control and organelle renewal, which can provoke a response to a series of stress conditions, such as starvation, ischemia, and many pathological stresses [40, 41]. Mitophagy, one of the selective forms of autophagy, can particularly remove dysfunctional mitochondria to keep mitochondrial homeostasis and cellular survivals [42]. During this procedure, mitochondrial dysfunctional is identified by the mitophagy receptors, consisting of BNIP3 (BCL2/adenovirus E1B 19 kDa interacting protein 3), NIX (BNIP3L), PINK1PINK1 (phosphatase and tensin homolog- (PTEN-) induced putative protein kinase 1), and Parkin and other related mediators [43]. Various studies have demonstrated that activating mitophagy to eliminate damaged and accumulated mitochondria can alleviate neuronal damage induced by cerebral ischemia/reperfusion injury [44–46]. Moreover, Chen et al. showed that a sphingosine kinase 2-mimicking TAT-peptide prevented neurons against ischemic injury by upregulating BNIP3-induced mitophagy [47]. Hypoxia inducible factor-1*α* (HIF-1*α*), a transcription factor, acts as the modulator of oxygen homeostasis, which is the upstream factor of BNIP3 [48]. It has been proved that BNIP3 overexpression can be mediated by HIF-1*α* in cerebral ischemia [49, 50]. Moreover, Xu et al. demonstrated that hypoxia-induced HIF-1*α* overexpression reduced the inhibition of dexamethasone on mitophagy via BNIP3 signaling pathway, thus suppressing apoptosis of bone cells [51]. Therefore, we hypothesize that HIF-1*α* plays a protective role by regulating BNIP3-mediated mitophagy, and TENS might regulate mitophagy through HIF-1*α*/BNIP3 pathway in ischemic stroke.

As for the selection of stimulation points, Baihui (GV20) and Hegu (LI4) are proved to be effective for the treatment of ischemic stroke [52]. Several studies have proved the neuroprotective roles of EA in MCAO models via stimulating at Baihui (GV20) and Hegu (LI4) points [53, 54]. Zhan et al. showed that EA induced anti-inflammatory effects by upregulating the zinc finger protein A20 expression via stimulating Baihui (GV20) and Hegu (L14) acupoints in MCAO rats [55]. In addition, Xie et al. reported that the EA stimulation in Baihui (GV20) and Hegu (L14) acupoints could improve the recovery of neurological function and mediate angiogenesis following ischemic stroke [56]. In clinical practice, these two acupoints were adopted to relieve post-stroke symptoms, including depression, anxiety, and insomnia [57–59]. Therefore, we selected the acupoints of Baihui (GV20) and Hegu (LI4) as the stimulating areas of TENS protocol.

Overall, in the present study, we aim to investigate the protective mechanisms of TENS intervention in the process of oxidative stress, neuroinflammation, and mitophagy in the acute stage of ischemic stroke.

## 2. Materials and Methods

### 2.1. Animals and Groups

All rats in this experiment were offered by Hebei Province Laboratory Animal Center. All adult male Sprague–Dawley (SD) rats, weighed 250–280 g, were raised in appropriate temperature (22 ± 2°C) with sufficient water and food in a 12 h light/dark cycle. The whole procedures of the present study were approved by the Animal Care and Use Committee of Hebei Medical University (ethical approval ID: 2021020). All protocols were abided by laboratory animal—guide for ethical review of animal welfare (GB/T 35892-2018) released in 2018 in China. The 75 SD rats were randomly divided into 3 groups (*n* = 25/group): (i) middle cerebral artery occlusion/reperfusion group (MCAO group), (ii) MCAO+TENS group (TENS group), and (iii) sham-operated group (sham group).

### 2.2. Middle Cerebral Artery Occlusion (MCAO) Model Establishment

The model of cerebral ischemia was established through blocking the middle cerebral artery (MCA). Before surgery, rats were fasted for 10 h but allowed to drink. The operation details are illustrated in our previous publications [17, 60].

### 2.3. Transcutaneous Electrical Nerve Intervention

The rats received TENS treatment with self-adhesive surface electrodes via a TENS apparatus (Model G6805-2A; Shanghai Huayi Co., Shanghai, China) after 3% sodium pentobarbital anesthesia. Remove the hair at the midpoint of the parietal bone (the position of Baihui acupoint, GV20) and the midpoint of radial border of the second metacarpal bone (the position of Hegu acupoint, LI4) and sterilize the skin with 75% ethyl alcohol. Then apply electrical stimulation to these two points for 30 min per day with a disperse-dense wave of 4 or 20 Hz, a current of 3-4 mA, and the intensity below the level of causing obvious muscle contraction. TENS was initiated at 24 h after MCAO/R surgery. After the treatment for three times (at 72 h following MCAO/R), the rats were sacrificed. Both sham and MCAO group only received the injection of 3% sodium pentobarbital without TENS intervention.

### 2.4. Assessment of Neurological Behaviors

The neurological deficit assessment was performed in all rats at 2 h or 72 h after MCAO/R in a blind manner. The details are reported in our published articles [16, 60].

### 2.5. TTC (2,3,5-Triphenyltetrazolium Chloride) Staining

This staining method was used to the detection of cerebral infarct volume. After sacrifice, the brain tissues from rats in all groups were obtained and frozen at -20°C for 15 min. The whole brain was then dissected coronally into 6 sections (2 mm every section) for TTC staining. The sections were soaked and stained in 2% TTC (TTC, Servicebio, China) at 37°C for 30 minutes. After 24 h to 48 h fixation in 4% paraformaldehyde buffer, photograph all slices. The region of infarction was calculated by Image J.

### 2.6. Measurement of Oxidative Stress Index

The brain tissues from cerebral cortex of the affected side in the rats (*n* = 5/group) were homogenized by RIPA buffer. Then collect the proteins. Levels of superoxide dismutase (SOD), malondialdehyde (MDA), glutathione (GSH), and glutathione peroxidase (GSH-px) were evaluated by commercially available kits (A001-3/A003-1/A005-1, Nanjing Jiancheng, Nanjing, China) in line with the manufacturer's protocol.

#### 2.6.1. ELISA

Based on the manufacturer's instruction, brain tissues were collected and lysed in lysis buffer. After homogenized ultrasonic treatment, the samples were centrifugated at 4°C, and the supernatant was obtained for ELISA. Read values of different groups at 450 nm with a microplate reader, and the concentrations of IL-6 and TNF-*α* were measured following the standard curve generated in the meantime.

### 2.7. Immunofluorescence

Immunofluorescence (IF) was conducted to analyze the expression difference of LC3 protein in the affected hippocampus of rats in three groups. Following anesthesia, five rats in each group were sacrificed. Brain tissues were removed and fixed in formaldehyde. After embedded in paraffin, the brains were sectioned into slices of 4 *μ*m thickness. The slices were blocked with the normal sheep serum at 37°C for 90 min and then immunostained with primary antibodies against LC3 (from rabbit; 1 : 300; 18725-1-AP, Proteintech Group, USA) at 4°C overnight. Next, the slices were incubated with Rhodamine- (TRITC-) conjugated goat antirabbit IgG (H + L) secondary antibodies (1 : 400, SA00007-2, Proteintech Group, USA) for 2 h in the darks. After washing 3 times with PBS, use 40, 6-diamidino-2-phenylindole (DAPI) to counterstain nuclei. Capture the images of hippocampus by a fluorescence microscope (OLYMPUS 905). Results were quantified as optical density (OD) by the Image J 1.8.0 program.

### 2.8. Western Blot

Western blotting was conducted to detect the expression of proteins related to apoptosis, oxidative stress, mitophagy, and neuroinflammation, including Bcl-2, Bax, TXNIP, GSDMD, caspase-1, NLRP3, BRCC3, HIF-1*α*, P62, and BNIP3. At 72 h after MCAO/R, rats (*n* = 5 each group) were euthanatized, and hippocampus in injuried hemisphere was collected and lysed as protein samples. The samples were subjected to SDS-PAGE to separate proteins and subsequently transferred onto polyvinylidene difluoride membrane (3010040001, Roche, Mannheim, Germany). After being blocked for 90 minutes with 5% bovine serum albumin, the membranes were put on the rotating mixer and incubated at 4°C for 16 h with the primary antibodies against BRCC3 (1 : 1000, Cat. #18215, Cell Signaling Technology, USA), TXNIP (1 : 1000, #14715, Cell Signaling Technology, USA), caspase-1 (1 : 2000, #ab184787, Abcam), NLRP3 (1 : 1000, #19771-1-Ig, Proteintech), GSDMD (1 : 500, #20770-1-AP, Proteintech Group, USA), HIF-1*α* (1 : 1000, ab179483, Abcam, UK), BNIP3 (1 : 300, ab109362, Abcam, UK), LC3 (1 : 1000; 18725-1-AP, Proteintech Group, USA), P62 (1 : 300, 66184-1-Ig, Proteintech Group, USA), Bcl-2(1 : 1000, #60178-1-Ig, Proteintech Group, Inc.), Bax (1 : 1000, #60267-1-Ig; Proteintech Group, USA), and GAPDH (1 : 5000, #60004-1-AP, Proteintech Group, USA). Following washing in in 0.1% Tween 20 in Tris-buffered saline (TBST) for 3 times, membranes were then incubated with HRP-conjugated secondary antibodies (1 : 5000, SA00001-1/SA00001-2, Proteintech Group, USA) for another 1.5 h at room temperature. Use the imaging analysis system of Amersham Imager 600 to make the protein bands visible with an ECL detection kit. At last, the optical density of these protein bands was calculated by Image J 1.8.0 (Scion Corporation).

### 2.9. Quantitative Real-Time PCR

Extract total RNA from brain tissues by TRIzol reagent (Life Technologies Corporation, Carlsbad, CA, USA). The concentrations and the quality of RNA were detected by a UV Spectrophotometer (N50 Touch; Implen, Germany). Quantitative Real-time PCR was applied using One-Step SYBR PrimeScript RT-PCR. The relative expression of mRNA was evaluated in line with 2^-ΔΔCt^ method normalized to GAPDH. The synthesis of primers was shown as follows: hsa-GAPDH: forward 5′- TGCACCACCAACTGCTTAGC-3′ and reverse 5′-GGCATGCACTGTGGTCATGAG-3′, has-NLRP3: forward.

### 2.10. Statistical Analyses

Make use of the statistic software, SPSS 21.0, to analyze all data. Differences among the multiple groups were conducted by one-way analysis of variance (ANOVA), followed by a Bonferroni test. The data from RT-qPCR and neurological deficits scores were performed by a nonparametric test. Quantitative values were expressed as mean ± standard deviation. *P* < 0.05 was considered as statistically significant.

## 3. Results

### 3.1. The Effect of TENS on Neuronal Pathological Changes after MCAO/R

**Figure 1:**
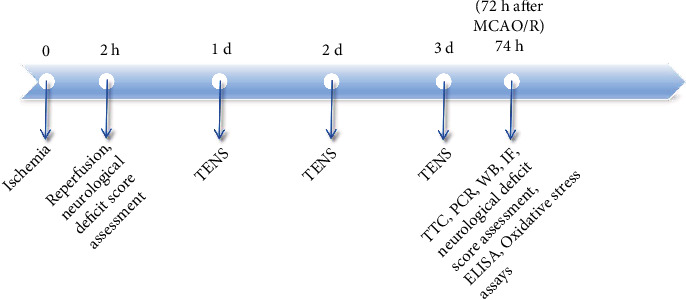
The experimental protocol at different time points.

**Figure 2:**
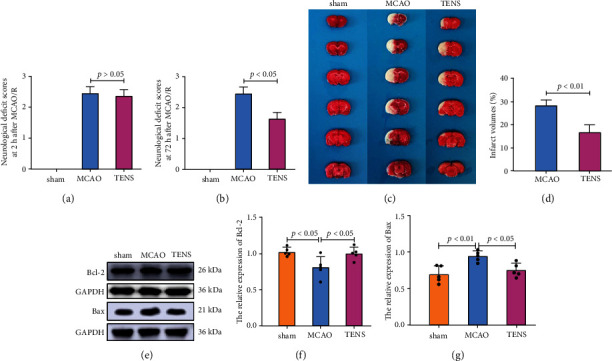
The pathological damage after cerebral ischemia/reperfusion (I/R) within 3 days. (a) Statistical analysis of neurological deficit score assessment at 2 h after MCAO/R injury. (b) Statistical analysis of neurological deficit score assessment at 72 h after MCAO/R injury. (c) The 2,3,5-triphenyltetrazolium chloride (TTC) staining demonstrating infarct volumes in MCAO rats with or without TENS. (d) Statistical analysis showing TENS significantly reduced neural damage when compared with the MCAO group. (e) Western blot analysis of the expression levels of Bcl-2 and Bax (GAPDH was used for the loading controls). (f, g) Statistical analysis showing the Bcl-2 and Bax expression in the injured hippocampus among three groups.

The neurological deficits were assessed at 2 h and 72 h after I/R surgery, as demonstrated in Figure 1. As shown in Figure 2(a), the sham group did not show any neurological symptoms, while the TENS group and MCAO group all showed distinct manifestations of neurological deficits. The TENS group exhibited no significant difference in neurological scores (Figure 2(a)) compared with the MCAO group at 2 h after MCAO/R operation (*P* > 0.05). However, in TENS group, neurological deficit scores were evidently reduced at 72 h compared with the MCAO group (*P* < 0.05, Figure 2(b)). Then, the infarction volume evaluation was implemented at 72 h following MCAO/R injury. The sham group showed no infarction. However, there was significant difference of infarct ratio between the TENS group and MCAO group (*P* < 0.05, Figures 2(c) and 2(d)). The infarcted volume of the TENS group was lower than MCAO group (*P* < 0.01). To further explore the mechanism of anti-apoptosis effects of TENS, as shown in Figures 2(e)–2(g), TENS treatment reversed the decrease of Bcl-2 (*P* < 0.05) and increase of Bax (*P* < 0.05) mediated by ischemic stroke.

### 3.2. Modulation of Oxidative Stress-Associated Proteins by TENS

**Figure 3:**
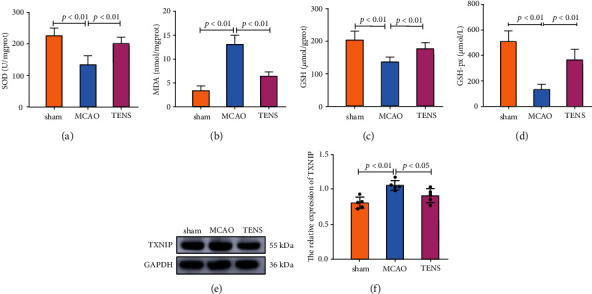
The effect of TENS treatment on oxidative stress-related proteins. (a–d) Statistical analysis of the SOD, MDA, GSH, and GSH-px activities assessed by kits. (e) TXNIP expression was detected by western blot. (f) Statistical analysis showing the TXNIP expression in the ipsilateral hippocampus among three groups.

As shown in Figure 3(b), ischemic stroke obviously increased MDA levels in the cortex (*P* < 0.01), and TENS significantly decreased MDA levels in the cortex (*P* < 0.01). Antioxidant enzymes were also measured. In Figures 3(a), 3(c), and 3(d), MCAO/R decreased the levels of SOD, GSH, and GSH-px compared with the sham group (*P* < 0.01); however, TENS restored the level of SOD, GSH, and GSH-px to some extent (*P* < 0.01). TXNIP level was low in sham group. TENS significantly suppressed the expression level of TXNIP (*P* < 0.05, Figures 3(e) and 3(f)).

### 3.3. Modulation of Neuroinflammation-Associated Proteins by TENS

**Figure 4:**
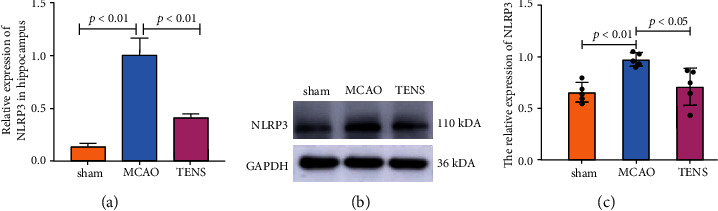
The effect of TENS treatment on NLRP3 expression. (a) NLRP3 expression in the injured hippocampus was detected by real-time PCR, in which GAPDH was used for the loading controls. (b) The NLRP3 expression was detected by western blot. (c) Statistical analysis showing the expression difference of NLRP3 and BRCC3 in the injured hippocampus among three groups.

**Figure 5:**
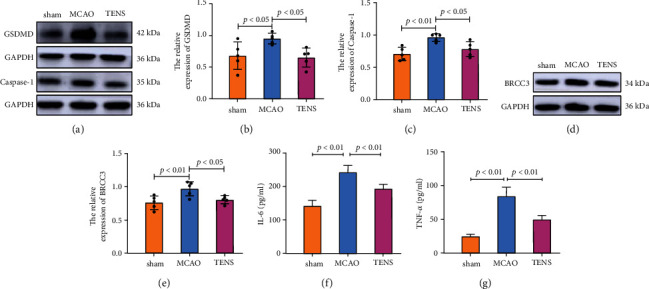
The effect of TENS treatment on neuroinflammation-related proteins. (a, d) The GSDMD, caspase-1, and BRCC3 expressions were detected by western blot. (b, c, e) Statistical analysis showing the expression difference of GSDMD, caspase-1, and BRCC3 in the injured hippocampus among three groups. (f, g) Statistical analysis of the levels of IL-6 and TNF-*α* assessed by ELISA kits.

As shown in Figure 4, MCAO/R induced the elevation of NLRP3 (*P* < 0.01), GSDMD (*P* < 0.05), and caspase-1 (*P* < 0.01), and TENS reversed the elevation in the affected hippocampus (*P* < 0.05), suggesting its anti-inflammatory effects (Figures 4(b), 4(c), and 5(a)–5(c)). Furthermore, BRCC3 expression was significantly raised in MCAO group (*P* < 0.01). TENS significantly inhibited the levels of BRCC3 (*P* < 0.05, Figures 5(d) and 5(e)). As shown in Figure 4(a), NLRP3 expression detected by RT-qPCR was increased in the MCAO group (*P* < 0.01). Furthermore, we verified that this elevation could be counteracted by TENS treatment (*P* < 0.01). In Figures 5(f) and 5(g), MCAO/R decreased the levels of IL-6 and TNF-*α* compared with the sham group (*P* < 0.01); however, TENS restored the increased level of IL-6 and TNF-*α* (*P* < 0.01).

### 3.4. Modulation of Mitophagy-Associated Proteins by TENS

**Figure 6:**
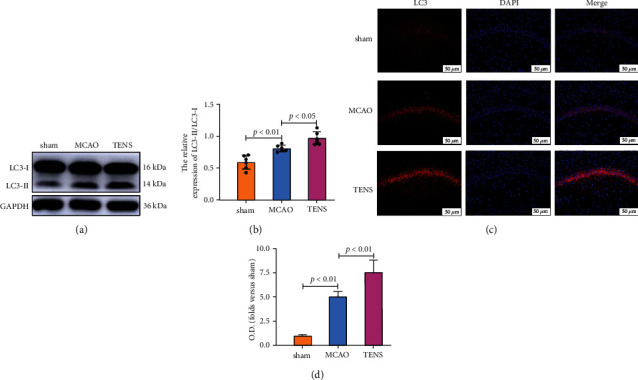
The effect of TENS treatment on LC3 expression. (a) The expression of LC3-II/LC3-I was measured by western blot, in which GAPDH was used for the loading controls. (c) IF analysis exhibiting the expression difference of LC3 protein in the ipsilateral hippocampus among three groups. (b, d) Statistical analysis of the expression of LC3 as assessed by IF.

**Figure 7:**
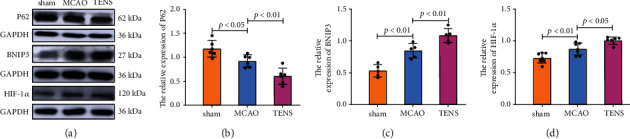
The effect of TENS treatment on mitophagy-related proteins. (a) The expressions of P62, BNIP3, and HIF-1*α* in the ipsilateral hippocampus were measured by western blot, in which GAPDH was used for the loading controls. (b, c, d) Statistical analysis of the expression difference of P62, BNIP3, and HIF-1*α* among three groups.

To explore the modulating mechanism of TENS in mitophagy after MCAO/R injury, the expressions of LC-3II/LC3-I, P62, BNIP3, and HIF-1*α* were evaluated by western blot. According to the Figures 6(a), 6(b), and 7(a)–7(d), the elevated expressions of LC-3 II/LC3-I (*P* < 0.01) and BNIP3 (*P* < 0.01) were detected in the MCAO group compared with the sham group, and their expressions were further increased in the TENS group (LC3-II/LC3-I, *P* < 0.05; BNIP3, *P* < 0.01). Also, the P62 levels were decreased in the MCAO group compared with the sham group (*P* < 0.05) and were further decreased in the TENS group than the MCAO group (*P* < 0.01). Moreover, HIF-1*α* was enhanced in the MCAO group compared with the sham group (*P* < 0.05), and its levels in the TENS group were even higher than the MCAO group (*P* < 0.05). As shown in Figures 6(c) and 6(d), the levels of LC3 as analyzed by immunofluorescence were also increased in the TENS group than the MCAO group (*P* < 0.05). These results indicate that TENS intervention can activate the cerebral ischemia/reperfusion-induced mitophagy potentially via the HIF-1*α*/BNIP3-dependent pathway.

## 4. Discussion

Globally, more than 16 million people are afflicted with stroke each year, leading to approximately one-third dying and another third becoming permanent disability, which causes the profound socioeconomic influence [61]. Moreover, previous studies confirmed that the prevalence and mortality rates of stroke were obviously higher in rural population compared with the urban population in China [62]. The rural population had the slower reduction of mortality in stroke than the urban population. These findings suggest that rural areas lag behind urban districts in socioeconomic and healthcare improvement [63]. Nowadays, despite great efforts and development in translational research, the available treatment strategies for stroke, including ischemic stroke, still remain limited, and numerous promising drugs in traditional preclinical evaluation are unable to enter clinical trials [63]. Therefore, a cost-effective and convenient treatment method is urgently needed, which could be important for rural and undeveloped areas.

Up to now, there are a growing number of interests toward the nonpharmacological approaches for improving stroke recovery due to the advantages of easy operation, availability, and economy, such as EA and electrical stimulation. The effects of EA on stroke have been widely studied in both clinical and experimental research [15, 64]. However, EA treatment is invasive with the risks of bleeding and infection, and some stroke patients cannot bear the pains of puncturing. In addition, the method of puncture relies heavily on the doctor's experience. In comparison, TENS is used widely in clinical practice because of its simplicity, good safety, and noninvasion [19]. Several clinical studies have reported the beneficial effect of TENS for poststroke patients, such as the improvement of motor, sensory dysfunction and dysphagia, and the symptomatic relief of shoulder hand syndrome, urinary incontinence, and constipation [65–71]. Currently, only the analgesic mechanism of TENS on multiple neuropathic pains is studied, including the electrical transmission modulation, the endogenous opioid peptides generation, and the blood vessel expansion [20, 72, 73]. However, the exact molecular mechanisms of TENS to treat stroke have not been investigated until now. Thus, in the present study, for the first time according to our knowledge, we demonstrated that TENS effectively decreased the neurological deficit scores, the cerebral infarction, and the levels of apoptosis in the acute stage of ischemic stroke, suggesting that TENS can be considered as a promising treatment strategy for ischemic stroke.

Furthermore, we investigated the exact mechanisms of neuroprotective effect of TENS on ischemic stroke. Following ischemic stroke, neuron injury was triggered by a series of pathological mechanisms, including oxidative stress, autophagy, neuroinflammation, and pyroptosis. Due to the disequilibrium between endogenous antioxidative system and detrimental ROS, ROS-triggered malondialdehyde (MDA) is evidently accumulated, and antioxidative enzymes (such as SOD and GSH-px) or antioxidant (including GSH) are reduced accordingly [74]. In this study, TENS treatment exhibited the antioxidant potential via promoting SOD, GSH, and GSH-px as well as inhibiting MDA. Moreover, TXNIP is an essential modulator for ROS signaling, which has been proved to accelerate cerebral ischemic injury [75]. In the current research, our results demonstrated that TXNIP expression was increased in the hippocampus following MCAO/R injury and conversely significantly reduced after TENS intervention, indicating the neuroprotective role of TENS.

Moreover, neuroinflammation plays a key role in the process of ischemic stroke [76, 77]. In innate immune system, NLRP3 inflammasome is a key node for immune sensing, readily responding to chronic inflammation. Moreover, NLRP3 can induce pyroptosis, one of the programmed cell death processes with a feature of plasm membrane damage, ensuing release of proinflammatory factors [35, 78]. The essential executor of pyroptosis is Gasdermin D (GSDMD). After being cleaved by caspase-1, GSDMD can form pores in cellular membrane, which causes cell death [79, 80]. Following ischemic stroke, NLRP3 inflammasome was reported to be activated, thus promoting neuron death [81]. Jiang et al. demonstrated that EA intervention inhibited NLRP3 inflammasome and subsequent inflammatory reaction via inhibiting caspase-1, GSDMD, and IL-1*β*, thus exerting neuroprotection against cerebral ischemic injury. Consistent with these findings, our results confirmed that TENS dramatically reduced the increased levels of proteins related to NLRP3 inflammasome-dependent pyroptosis and neuroinflammation after ischemic stroke, consisting of NLRP3, GSDMD, caspases-1, IL-6, and TNF-*α*. Moreover, as a regulator of the ubiquitination and activity of NLRP3, BRCC3 was activated following cerebral ischemia and TENS treatment reversed the promoted BRCC3 levels induced by ischemic condition. These results suggested that TENS may effectively alleviate brain damage against ischemic stroke via suppressing NLRP3 inflammasome activation by regulating BRCC3.

Mitophagy contributes to the physiology of organelle integrity and cellular energy by maintaining the mitochondria biogenesis and homeostasis, which is often considered as the essential mechanism for cell survival [82]. Rapamycin-activated mitophagy was proved to improve mitochondrial function through decreasing malondialdehyde (MDA) and recovering the levels of ATP and mitochondrial membrane potential in the rat models of cerebral ischemia [83]. Moreover, Zhong et al. demonstrated that EA improved cognitive impairment by inhibiting NLRP3 inflammasome activation by upregulating mitophagy induced by melatonin in stroke models [84]. Consistently, in the present study, the expression of BNIP3 and the ratio of LC3-II/I was obviously enhanced, and P62 expression was significantly reduced after MCAO/R injury. TENS treatment further elevated the expression level of BNIP3 and LC3-II/I and reduced the P62 expression. These results indicated that BNIP3-mediated mitophagy was activated following cerebral ischemia, and TENS could exert neuroprotective effects by activating mitophagy. HIF-1*α* plays a key role in adaption to hypoxic and ischemic conditions, which modulates the expression of targeted proteins involved in apoptosis and oxidative stress, particularly mitophagy [85–87]. Liu et al. showed that the expression of HIF-1*α* was increased at 72 h following MCAO surgery [88]. Furthermore, the upregulation of mitophagy induced by activated HIF-1*α*/BNIP3 signaling pathways was proved to inhibit apoptosis in acute kidney injury [89]. In our present study, we showed that HIF-1*α* was activated following I/R injury, and TENS treatment markedly promoted the HIF-1*α* expression and increased BNIP3-mediated mitophagy levels, thus indicating that HIF-1*α* and BNIP3 might participate in the regulation mechanism of TENS treatment for ischemic stroke.

There are several limitations in this research. First, our study focuses on the neuroprotection of TENS against ischemic brain injury, without identifying the different effects among different stimulation sites of TENS. In the future study, we will set different control groups to explore the difference between the stimulated acupoints of “Baihui and Hegu” and other stimulation acupoints. Second, we did not perform rescue experiments with the inhibitors or inducers of autophagy, inflammation, TXNIP, BRCC3, and HIF-1*α*. Further studies are needed to confirm the involvement of autophagy, inflammation, TXNIP, BRCC3, and HIF-1*α* in the neuroprotective mechanisms of TENS for alleviating cerebral ischemic injury.

## 5. Conclusion

Taken together, the current results demonstrate that TENS significantly modulates oxidative stress, neuroinflammation, pyroptosis, and neuronal autophagy following ischemic stroke, possibly via regulating TXNIP, BRCC3/NLRP3, and HIF-1*α*/BNIP3 signaling pathway, thus reducing the brain damage caused by cerebral ischemia. However, the specific mechanisms for the neuroprotective effect of TENS on ischemic stroke remain to be further explored.

In summary, TENS treatment might be a rational and cost-effective strategy for improving patient function following ischemic stroke and deserves further exploration, thus benefiting more stroke patients.

## Data Availability

The data used to support the findings of this study are available from the corresponding author upon request.
